# Editorial: *Mycobacterium abscessus*; The Paradox of Low Pathogenicity and High Virulence

**DOI:** 10.3389/fmicb.2022.943694

**Published:** 2022-06-10

**Authors:** Thomas F. Byrd, Edward D. Chan

**Affiliations:** ^1^School of Medicine, University of New Mexico Health Sciences Center, Albuquerque, NM, United States; ^2^Division of Infectious Diseases, Department of Internal Medicine, University of New Mexico, Albuquerque, NM, United States; ^3^National Jewish Health, Denver, CO, United States; ^4^Department of Medicine, Division of Pulmonary Sciences and Critical Care Medicine, School of Medicine, University of Colorado, Aurora, CO, United States; ^5^Rocky Mountain Regional VA Medical Center, VA Eastern Colorado Health Care System, Aurora, CO, United States

**Keywords:** *Mycobacterium abscessus*, cystic fibrosis, porin, biofilm, glycopeptidolipid

*Mycobacterium abscessus* is a non-tuberculous mycobacteria that is an important cause of progressive human pulmonary infection. While it is a bona fide cause of lung and extra-pulmonary disease, its potential to do so is relatively low. Indeed, *M. abscessus* pulmonary infection does not typically occur in immunocompetent individuals with normal lung airways. On the other hand, individuals with abnormal lung airways who are otherwise immunocompetent can develop progressive, severe, intractable pulmonary infection. As further evidence of its low pathogenicity, significant experimental modifications in the study design are often required in order to establish a productive infection in mice; i.e., use of various types of immunodeficient mice is typically needed to create an established infection. The lack of suitable mouse models that reproduce abnormal lung airways seen in humans further hampers these efforts. One important aspect of *M. abscessus* pathogenesis is colonization of abnormal lung airways with glycopeptidolipid (GPL)—expressing smooth colony variants that are able to form biofilms. Spontaneous emergence of virulent rough colony variants lacking GPL leads to invasive lung infection and resultant increased lung inflammation (Byrd and Lyons, 1999; Howard et al., 2006; Catherinot et al., 2007, 2009; Nessar et al., 2011).

The articles in this Research Topic describing *M. abscessus* biofilm formation in synthetic medium (Belardinelli et al.) and increased virulence of outer membrane porin mutants of *M. abscessus* (de Moura et al.) provide evidence for modulation of *M. abscessus* pathogenicity by selective pressure within the lung airways of cystic fibrosis patients. These studies focus on the evolving pathogenic sequence of *M. abscessus* lung infection when the low pathogenic colonizing lung airway phenotype is transitioning to its more virulent, proinflammatory phenotype. The biofilm study elucidates the genetic and biochemical differences between *M. abscessus* subsp. *abscessus* grown as biofilm in minimal synthetic medium to the same organism grown in an enriched medium designed to mimic the conditions *M. abscessus* may encounter in the lung airways of cystic fibrosis patients (synthetic cystic fibrosis medium—SCFM). Among the differences noted was the significant presence of GPLs in the extracellular matrix of biofilms formed by *M. abscessus* in SCFM, highlighting the important role that these cell wall components play in *M. abscessus* biofilm formation. Extracellular DNA was also present in relative abundance in the biofilms matrix formed in SCFM and provides support for the established use of recombinant human DNase treatment in patients with cystic fibrosis. These results indicate that growth of *M. abscessus* biofilm in SCFM is a useful method for identifying potential virulence determinants upregulated under conditions that mimic the airways of cystic fibrosis patients. Similarly the porin paper provides evidence that deletions and rearrangements in porin genes of serially collected *M. abscessus* isolates from cystic fibrosis patients occur in response to host selection pressure. Specifically, deletion of two of these porin genes, *mmpA* and *mmpB* leads to enhanced replication of the knock out mutants in bone-marrow derived mouse macrophages from SCID and GM-CSF knock-out mice, as well as in a SCID mouse model of infection. Findings from both these studies support the concept that host selection pressures enhance *M. abscessus* virulence once colonization of lung airways occurs.

One hypothesis for the establishment of *M. abscessus* lung infection is that chronic inflammation results in suppression of the Th1 immune response needed for clearance (Orme and Ordway, 2014). If this is the case, the paper describing the effects of Rufomycin directly on *M. abscessus* and the host immune response (Park et al.) suggests a dual mechanistic strategy for treatment of *M. abscessus* lung infection using this drug. By both directly inhibiting the organism and blunting the proinflammatory response elicited by rough invasive variants, this drug may act in a novel way to prevent this bacterium from achieving its full virulence potential. The paper describing an interferon-gamma release assay (IGRA) (Steindor et al.) demonstrates the potential use of an *M. abscessus* specific antigen in an IGRA designed to diagnose *M. abscessus* infection. The question of what is the correlate of an effective immune response to *M. abscessus* lung infection remains open. In this study, the majority of patients were cystic fibrosis patients with only a small number of non-CF bronchiectasis patients. Additional studies using the assay would be informative in distinguishing the IGRA response in patients with chronic lung airway colonization compared to those with invasive parenchymal lung disease as well as in those patients colonized/infected with smooth GPL expressing isolates compared to rough isolates lacking GPL.

Together these articles are important contributions to the *M. abscessus* field, and provide evidence that supports the concept that *M. abscessus* has low pathogenicity until it is able to colonize its preferred niche in abnormal lung airways leading to enhanced virulence, invasive lung infection and avoidance of host immune defense mechanisms.

## Author Contributions

TB and EC contributed to the development of the idea for this Research Topic, solicited manuscripts, and contributed to the final version of the Editorial.

## Conflict of Interest

The authors declare that the research was conducted in the absence of any commercial or financial relationships that could be construed as a potential conflict of interest.

## Publisher's Note

All claims expressed in this article are solely those of the authors and do not necessarily represent those of their affiliated organizations, or those of the publisher, the editors and the reviewers. Any product that may be evaluated in this article, or claim that may be made by its manufacturer, is not guaranteed or endorsed by the publisher.
